# Fiber Bragg Grating Monitoring of Full-bolt Axial Force of the Bolt in the Deep Strong Mining Roadway

**DOI:** 10.3390/s20154242

**Published:** 2020-07-30

**Authors:** Peng Wang, Nong Zhang, Jiaguang Kan, Zhengzheng Xie, Qun Wei, Wenhao Yao

**Affiliations:** 1 Key Laboratory of Deep Coal Resource Mining of the Ministry of Education, School of Mines, China University of Mining and Technology, Xuzhou 221116, China; wangpeng19@cumt.edu.cn (P.W.); jgkan@126.com (J.K.); xie_zz@cumt.edu.cn (Z.X.); weiqun@cumt.edu.cn (Q.W.); TS17020074A3ZJ@cumt.edu.cn (W.Y.); 2 Open Laboratory for Large-Scale Scientific Instruments, Jiangsu Normal University, Xuzhou 221116, China

**Keywords:** fiber bragg grating, full-bolt, axial force, deep strong mining, real-time monitoring

## Abstract

With the increase of mining depth and strength, the evolution of bolt axial force is increasingly becoming important for ensuring the reliability and safety of support. To improve the problem of the existing coal mine roadway pressure-monitoring method, whereby it is difficult to continuously monitor the axial force of the bolt over a long period of time, a full rod fiber bragg grating (FBG) force-measuring bolt and system were designed based on the principle of fiber grating sensing. It was found that a trapezoidal groove is a relatively better groove. The linearity between the center wavelength offset of the fiber grating and the axial force was more than 0.99, and the conversion formula between the axial force of the bolt rod and the wavelength change of the fiber grating were obtained. The real-time monitoring revealed that the axial force of the bolt obviously changed before and after compression. The axial force distribution curve can be divided into the stable zone, growth zone, and peak zone. The influence of the roadway abutment pressure was approximately 130 m ahead of the working face, and the peak area was within the 25–35 m range of the advance working face. The axial force of the bolt rod at the end of the anchorage linearly increased with the tail end of the bolt, the axial force of the free segment was the largest, and the overall stress was essentially the same. The application results demonstrate the feasibility and effectiveness of the FBG full-length force bolt.

## 1. Introduction

The bolt support method is safe and efficient and is also the primary support method used in China’s coal mine roadway. This is an active support method, and it provides timely support resistance to the surrounding rock and effectively improves the surrounding rock strength to restrain the separation and deformation of the surrounding rock [1,2,3]. Deep mining roadways face many factors such as high ground stress, high ground temperature, high osmotic pressure, and mining disturbances, which are challenging for bolt support [4,5,6]. Therefore, to increase the reliability of bolt support and roadway safety, it is becoming increasingly important to monitor the law governing the bolt’s axial force evolution by means of refinement.

There are two traditional methods for monitoring the axial force of a bolt: one is to stick the resistance strain gauge on the surface of the rod, use the synchronous deformation of the rod and strain gauge to drive the resistance of the strain gauge, and then reverse the law governing the change of the bolt’s stress and strain. The other is to test the pull-out force and axial force of the bolt cable using a pull-out instrument and dynamometer or test the pull-out force and axial force of the bolt cable using a hydraulic pillow [7,8]. However, the above-mentioned methods have various problems such as insufficient accuracy and timeliness, and cannot realize real-time monitoring, thereby introducing potential safety risks to coal mine production. As a new sensing technology, fiber Bragg grating (FBG) has attracted the attention of scholars in many fields, including the mining industry, owing to its intrinsic safety, corrosion resistance, electromagnetic interference, and distributed sensing [9,10,11].

Hill et al. [12] first discovered the photosensitive effect of optical fiber in germanium-doped silica fiber and made the first optical fiber grating in the world using the standing wave writing method. Presently, FBG is widely used in many fields such as military security, petrochemical, geotechnical engineering, aerospace, and electric power, and has achieved remarkable results. Meltz et al. [13] of Brown University in the United States first proposed an optical fiber sensor for reinforced concrete structure monitoring. Xu et al. [14] reviewed the application of FBG sensors in various engineering fields. Jiang et al. [15] established an FBG bolt cable pre-stress monitoring system, and preliminarily realized the pre-stress detection of a bridge bolt cable. Lin et al. [16] applied the FBG sensor to the axial force monitoring of the Longtan Tunnel support and obtained the change characteristics of the bolt axial force under this condition.

Various attempts have also been made in the coal industry and some applications have been introduced. Chai et al. [17] used fiber grating sensing technology to investigate the settlement and deformation of the overlying strata in a test slope. Fang et al. [18] developed the working face support pressure gauge and tested its performance based on the principle of the FBG sensor. Zhao [19] designed a type of FBG force-measuring bolt, applied it to the 11,155(1) working face of Guqiao Mine, and measured the axial force evolution of the bolt within a certain period of time. Wang et al. [20] investigated the difference of the axial force distribution between the end anchorage and full-length anchorage in the laboratory using the FBG sensor. Ho et al. [21] proposed an FBG intelligent bolt plate to monitor the force of bolt ends. As can be seen, in the coal industry, FBG monitoring has mostly been used in laboratory testing. Field applications have mostly focused on the axial force monitoring of the bolt end section. The axial force monitoring of the bolt rod in different positions in a deep mine under high-stress conditions has not been extensively investigated to date, there is a lack of coupling correlation analysis between in situ monitoring data and ground pressure behavior characteristics in the severe environment of deep mine strong mining roadway (Load of bolt—Surrounding rock deformation—Stress evolution), and the influence of slotting on the rod strength under high confining pressure has also been poorly investigated.

According to previous research results and experience, in this study, the stress and strain of different groove-shaped bars under high confining pressures were investigated using the finite element analysis software ANSYS [22]. A full-length distributed FBG force-measuring bolt and its installation method were designed, and a calibration test was conducted for the force-measuring bolt. The sensitivity and other correlation coefficients of this type of bolt material were obtained, and the wavelength axial force conversion formula was determined. The FBG full rod axial force real-time monitoring system was applied to the entire process of mine pressure monitoring in a deep well and strong mining roadway. The monitoring results reveal that the system has good performance, feasibility, and effectiveness.

## 2. Principle of FBG Sensing

**Figure 1:**
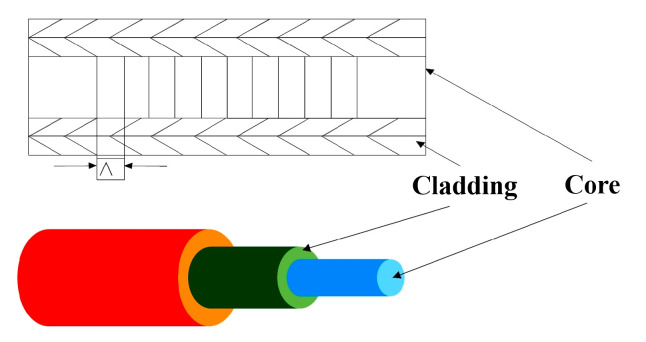
Optical fiber structure.

Optical fiber is a type of dielectric waveguide working in optical wave band [23]. Its basic structure is cylindrical, and comprises an optical fiber core, optical fiber cladding, optical fiber protection layer, reinforced fiber, and optical fiber protection sleeve, as shown in Figure 1.

**Figure 2:**
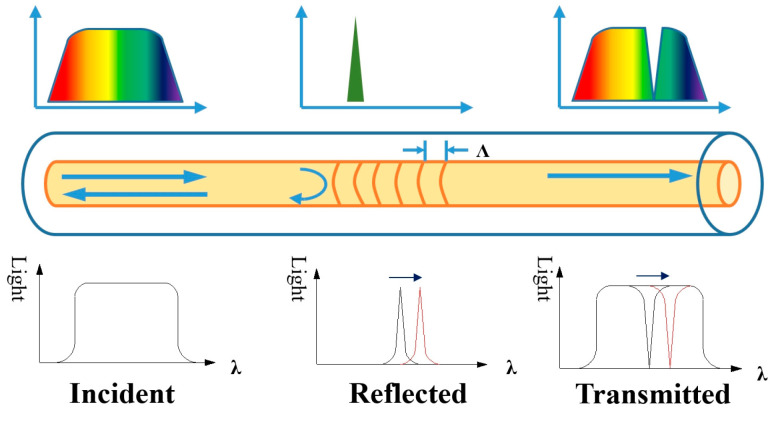
Schematic diagram of fiber Bragg grating sensing.

Fiber grating is a section of the fiber with a periodic refractive index change formed by ultraviolet (UV) etching in the fiber core. As shown in Figure 2, when the broadband light source is transmitted in the fiber core, the incident light wave is reflected back in a specific band, and most of the remaining light continues to be conducted along the original direction in the form of a transmitted light wave. In the spectrum of the transmitted light wave, a part of the narrow band spectrum with the reflection wavelength at the center is missing, which plays the role of light wave selection [24,25].

When the λ of the incident light of the fiber satisfies the Bragg diffraction condition, the expression is as follows [26]:

(1) $$\lambda_{B}=2n_{eff}\Lambda ,$$

where *λ_B_* is the center peak wavelength of fiber Bragg grating; *n_eff_* is the refractive index of the effective fiber core; and Λ is the grid distance between adjacent gratings, which is called the period of fiber gratings.

The period Λ and effective refractive index of the fiber grating can be changed by pulling and pressing the fiber grating or changing the temperature. Therefore, the reflection wavelength of the fiber grating can be changed, which causes the wavelength drift of the fiber grating. Hence, the fiber grating can be connected with the mine pressure monitoring system, and a variety of sensors can be made to establish a real-time online monitoring system based on modern sensing technology.

## 3. The Design of FBG Force-Measuring Bolt

### 3.1. Comparison of Strength and Damage of Different Groove-Type Bolts Under High Confining Pressure

#### 3.1.1. Method

In bolt supports, the strength of the rod greatly influences the support effect. In the design process of the force-measuring bolt rod, slotting will destroy the integrity of the rod body, which has a certain impact on the strength of the rod. To investigate the influence of different slotting shapes on the strength of the rod, the ANSYS finite element analysis software is an effective simulation method, and can assess the strength and damage of the materials by the stress concentration and strain of the materials under stress. Zhao et al. [27] used the static analysis module of the ANSYS software to investigate the strength of different groove-shaped bars without in situ stress. However, in the process of deep mining, the characteristics of “three highs and one disturbance” are particularly obvious, and the analysis of the strength of the rod under high ground pressure and disturbance stress is insufficient. Therefore, in this study, using sketch module and static structure module of the ANSYS software, the bolt element was established to simulate the strength damage of different groove bars under high ground pressure.

#### 3.1.2. Simulation Scheme

To investigate the stress–strain evolution characteristics of different groove type bolt rods under high confining pressure, four sets of numerical models were established: no groove, rectangular groove, trapezoidal groove, and inverted trapezoidal groove. The simulation material is isotropic steel, and the specific parameters of the simulated bolt are: length of 50 mm, diameter of 20 mm, rod density of 7850 kg/m^3^, Young’s modulus of 20.9 GPA, Bulk’s modulus of 15.1 GPA, Shear’s modulus of 8.2 GPA, Poisson’s ratio of 0.269, and mesh size of 0.5 mm.

**Figure 3:**
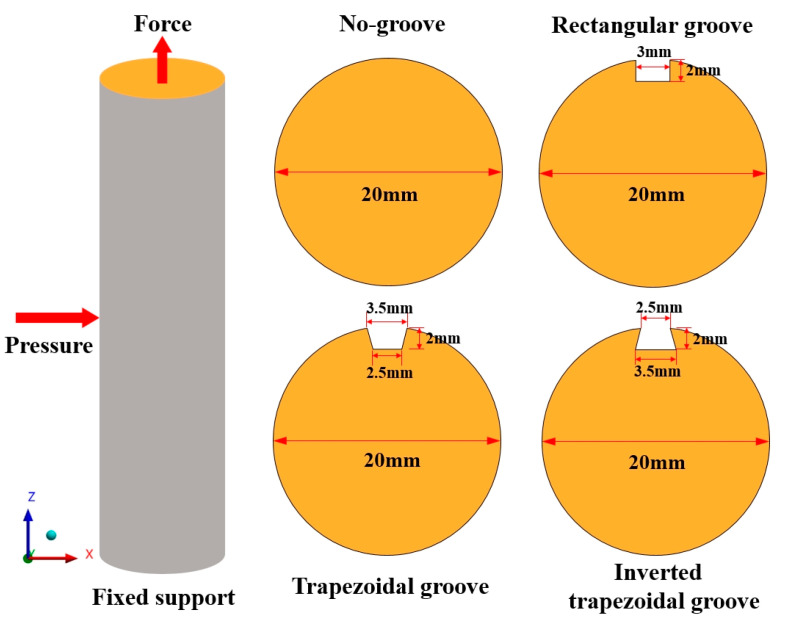
Schematic diagram of different groove types and boundary conditions.

Because the bolt is surrounded by rock in the supporting state, and bears a large confining pressure in the mining state of a deep well, to better restore the stress state of the underground bolt, a confining pressure of 25 MPa (buried depth of 1000 m) was applied to the rod body in the simulation process, the bottom surface was completely fixed, the top surface was free, and a tension of 120 KN was applied. The boundary conditions were x and y direction displacement constraints, and the boundary conditions and groove size are shown in Figure 3.

#### 3.1.3. Simulation Results and Analysis

**Figure 4:**
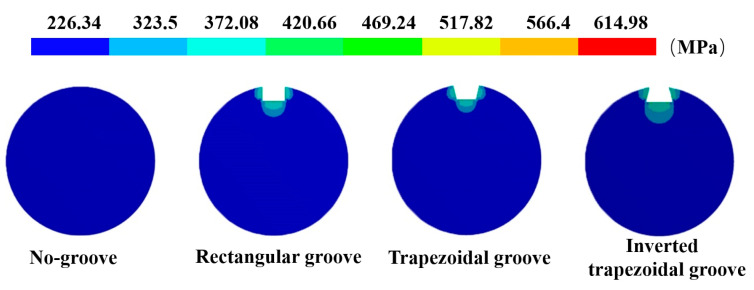
Stress distribution of different grooves.

The stress profile of the cross section of the rod body under different groove types in the high confining pressure state is shown in Figure 4. After the rod body was slotted, a stress concentration phenomenon appeared around the groove under the action of tension and confining pressure. Compared with that without confining pressure, the stress peak value significantly increased and the displacement of the rod body slightly increased. When there was no groove, the stress of the rod body was 241.42 MPa, and the displacement of the rod body was 0.733 mm. The maximum stress of the rod body with a rectangular groove was 603.2 MPa, the concentration factor was 2.5, and the displacement of the rod body was 0.759 mm. The maximum stress of the rod body with a trapezoidal groove was 590.29 MPa, the concentration factor was 2.44, and the displacement of the rod body was 0.757 mm. The maximum stress of the rod body with an inverted trapezoidal groove was 614.98 MPa, the concentration factor was 2.55, and the displacement of the rod body was 0.761 mm. According to the comparison of stress and displacement, the inverted trapezoidal groove had the greatest influence on the rod strength, while the trapezoidal groove had the least influence on the rod strength.

### 3.2. Structure and Installation of FBG Force-Measuring Bolt

The FBG force-measuring bolt mainly consists of five parts: a metal rod, FBG, optical fiber, jacking nut, and installation joint. The rod was selected from the underground tunnel support construction bolt.

The bolt size was Φ20 × 2400 mm. According to the above-mentioned simulation results, the groove type was trapezoidal with a width of 3.5 mm at the top, width of 2.5 mm at the bottom, and depth of 2 mm. There existed a total of 10 gratings arranged on the entire rod. Here, the purpose was to monitor the axial force of the entire rod in the anchorage segment and free segment. The length of the anchorage segment was 700 mm, the length of the free segment was 1650 mm, and the exposed length was 50 mm. The gratings were unevenly arranged, with five gratings in the anchorage segment and free segment. The spacing of the grating in the anchorage segment was 150 mm, and the positions were 50 mm, 200 mm, 350 mm, 500 mm, and 650 mm from the tail of the anchorage end. The spacing of the grating in the free segment was 300 mm, and the positions were 800 mm, 1100 mm, 1400 mm, 1700 mm, and 2000 mm from the tail of the anchorage segment.

**Figure 5:**
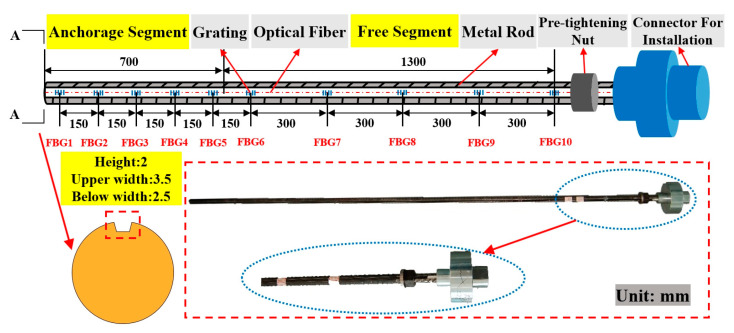
Structure of fiber bragg grating (FBG) force-measuring bolt.

The bolt rod manufacturing process is divided into the following steps: first, the bolt rod surface is treated, the polishing and leveling process is completed with a grinder and sandpaper, and the oil and dust are wiped to avoid the pollution of the grating. Subsequently, the grating sensors were arranged in groups along the groove axis using 502 glue such that the grating sensors and rod surface were as close as possible to ensure the accuracy of the monitoring data. Finally, the FBG and bolt groove were sealed with resin glue, which can prevent the sensor from being damaged during the bolt installation and provide moisture-proofing and buffer protection. Figure 5 shows the specific schematic diagram and bolt after the completion of encapsulation.

### 3.3. Real-Time Monitoring System for Axial Force of the Entire Rod

The entire rod axial force real-time monitoring system comprises a flameproof fiber grating signal processor, FBG force-measuring bolt, jumper, grating junction box, and optical cable.

**Figure 6:**
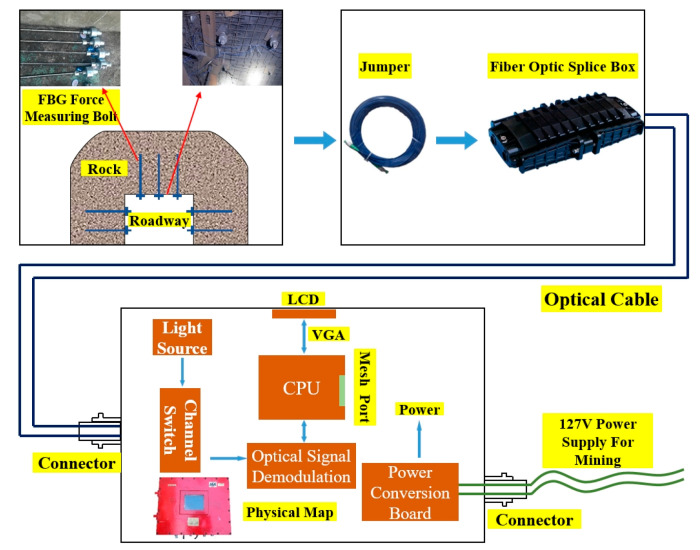
Hierarchical structure of the monitoring system.

The signal processor shell uses an explosion-proof shell, built-in 127-V mining power conversion circuit, optical signal adjustment module, display module, channel adjustment module, and optical fiber transceiver. Each grating installed in the bolt body reflects a specific wavelength. When the surrounding rock of the tunnel is active, the bolt is subject to axial tension deformation, and the grating embedded in the rod body and the rod body itself are synchronously deformed and transmitted in the form of a light wave in the optical fiber. The real-time monitoring system can demodulate the change of the axial force of the bolt by analyzing and processing the change of the grating wavelength and realizing the visualization of the bolt’s axial force state. The entire system is shown in Figure 6.

For the underground installation of the FBG force-measuring bolt, the construction process of ordinary bolt supports is used. A Φ30 mm drill bit is used to drill holes with a hole depth of 2360 mm. Then, a msk2370 resin anchoring agent is added, and a mixer is used to connect the installation joint with the bolt drill. The bolt is raised to the top nut and tray, stuck to the coal wall, mixed for 20–25 s, and then the machine is stopped. After waiting for 90 s, the secondary fastening is completed. After the bolt is installed, the back cover of the installation joint is opened, the jumper, grating junction box, and processor are connected and the power is turned on to realize the real-time monitoring of the axial force for the entire bolt.

## 4. Wavelength-Axial Force Conversion

### 4.1. Experimental Equipment and Materials

To ensure the accuracy of the field test monitoring data, it is necessary to calibrate the FBG force-measuring bolt to determine the sensitivity and other correlation coefficients and the calculation formula of the bolt axial force.

The calibration system consists of a main frame, force application device, and fiber-grating wavelength acquisition and recognition system. The main frame comprises a fixed frame, fixed plate, baffle, calibration frame, and pre-tightening nut. The force application device is connected with a drawing jack, which can provide an axial force of 0–100 KN. The FBG wavelength acquisition and recognition system adopts the FBG signal demodulator produced by the Paishuo intelligent technology company in Shanghai. The main technical indicators are: 10 channels, scanning frequency of 0–100 Hz, wavelength range greater than 1520–1570 nm, peak reflectivity of 99%, and temperature of 27 °C.

**Figure 7:**
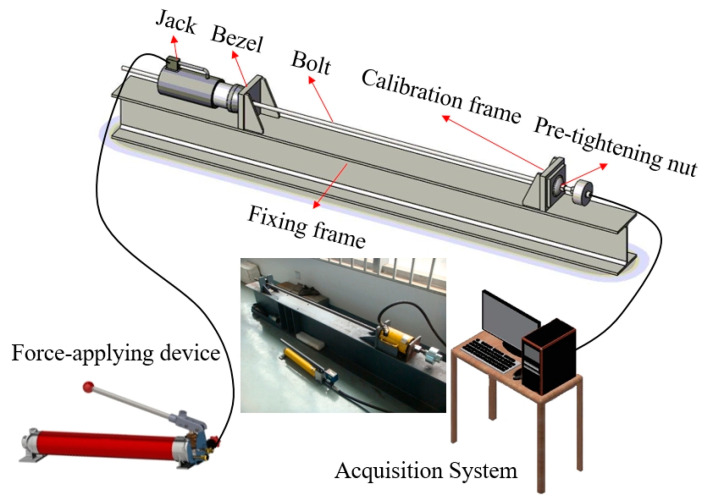
Calibration system of FBG force measuring bolt.

The calibration material is the packaged FBG force-measuring bolt. The calibration process is illustrated in Figure 7.

### 4.2. Experimental Scheme

- The FBG force-measuring bolt was fixed onto the calibration frame, with a fixed plate at one end of the installation joint. The jack at the end of the rod body was pulled out, and a baffle plate was used for tightening.
- Axial tension was applied to the drawing jack by the force application device, and the axial force change and grating wavelength signal change of the force application device were transmitted to the acquisition and recognition system and recorded.
- During the experiment, the method of equal gradient axial force loading was adopted. A jack was used to apply pressure of 0, 15 KN, 30 KN, 45 KN, and 60 KN to the force-measuring bolt. Five loading tests were conducted in total. The wavelength values of 10 gratings under different axial forces were recorded. The relationships between the wavelength change of the force-measuring bolt and the different axial forces were compared.

### 4.3. Analysis of Experimental Results

#### 4.3.1. Linear Regression Fitting

Under the action of uniform strain (constant temperature), the expression of the reflection wavelength change of the fiber grating is as follows:

(2) $$\Delta \lambda_{B}=\lambda_{B}\left(1-p_{e}\right)\varepsilon_{grating},$$

(3) $$\varepsilon_{grating}=\frac{\Delta \lambda_{B}}{\lambda_{B}\left(1-p_{e}\right)},$$

where *p_e_* is photoelastic coefficient; *ε_grating_*. The grating is the axial strain of the grating; and Δ*λ_B_* is the center wavelength offset of the fiber grating.

When the FBG sensor and the bolt were encapsulated together, the axial dimension of the grating was very small. Hence, it is assumed that the axial strain of the bolt is the same as that of the FBG sensor [28].

(4) $$\varepsilon_{grating}=\varepsilon_{bolt},$$

According to Equations (3) and (4), the axial force of the bolt can be expressed as follows:

(5) $$\sigma =E\varepsilon =\frac{E\Delta \lambda_{B}}{\lambda_{B}\left(1-p_{e}\right)},$$

Available bolt axial force *F*:

(6) $$F=\sigma A=\frac{EA\Delta \lambda_{B}}{\lambda_{B}\left(1-p_{e}\right)},$$

where *F* is the axial force of bolt, N; *E* is the elastic modulus of bolt, MPa; and *A* is the cross-sectional area of bolt, m^2^.

When the axial force of the bolt was not large, the change of its cross-sectional area was also very small and could be ignored. Therefore, *A* can be considered as constant. After the bolt material was determined, its elastic modulus *E* remained unchanged. Therefore, the axial force *F* of the bolt at a certain point is linearly related to the drift of the center wavelength of the FBG at that point.

**Figure 8:**
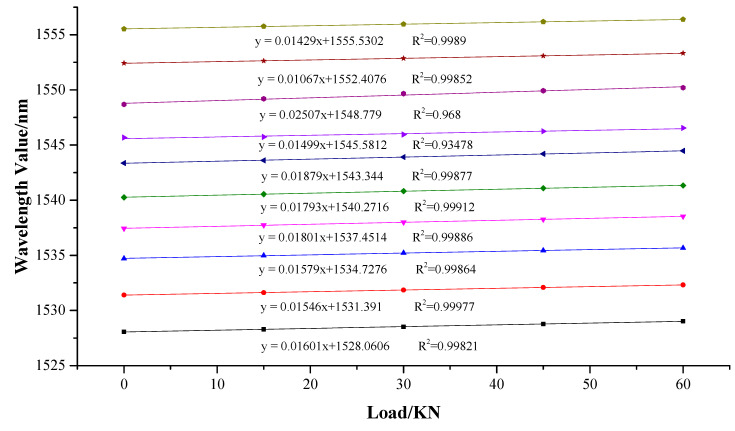
Fitting analysis of loading test results.

From the calibration data in Figure 8, it can be seen that, with the increase of the applied axial force, the wavelength values of channels 1 to 10 on the FBG force-measuring bolt exhibit an increasing trend. Through linear regression fitting between the wavelength values of 10 FBGs and the applied axial force data, the corresponding fitting equation was obtained. Thus, it is concluded from the figure that the linear fitting degree of 10 gratings is essentially greater than 0.99, and the maximum is 0.999, which is close to 1. This indicates that the linear fitting degree was very high, which is essentially consistent with the theoretical derivation. There are two grating linear fitting degrees of 0.93 and 0.968, which are relatively low. The analysis reveals that various errors exist, mainly owing to the sticking deviation in the process of making the force-measuring bolt rod, which makes the test results appear erroneous. However, this will gradually improve in future tests.

#### 4.3.2. Calculation of Bolt Axial Force

Considering the difference between the grating sensitivity and temperature sensitivity in different channels, the formula of the bolt axial force is deduced by taking the axial force of the bolt as the independent variable and the grating wavelength as the dependent variable. The calculation formula of the axial force of the bolt considering the temperature compensation is expressed as follows:

(7) $$\lambda_{i}=(GF_{i}+\lambda_{0})+K(T_{i}-T_{0}),$$

where:

$\lambda_{i}$ is the output wavelength value of the sensor at time *i*, nm;
*G* is the sensor sensitivity coefficient, nm/KN;
$F_{i}$ is the axial force on the bolt at time *i*, KN;
*K* is the temperature correction coefficient of the sensor, nm/°C;
$T_{i}$ is the temperature value at time i, °C;
$T_{0}$ is the original grating temperature, °C.

Therefore, we get:

(8) $$F_{i}=\frac{\lambda_{i}-K(T_{i}-T_{0})-\lambda_{0}}{G},$$

After the completion of bolt encapsulation, the sensitivity coefficient of each channel is calibrated, the original wavelength value is recorded, and the real-time wavelength value is obtained based on an acquisition and recognition system. Then, the axial force of the bolt at this time can be calculated. Because the temperature of underground roadway in coal mine is relatively constant, especially when the bolt is constructed into the surrounding rock, the temperature is basically unchanged. Therefore, the compensation of the axial force of the FBG force-measuring bolt can be ignored. In addition, considering construction damage and cost issues, no temperature compensation bolt is set, so the calculation is directly based on the difference between the collected wavelength and the original wavelength.

## 5. Engineering Application

### 5.1. Engineering Background

The Kouzidong coal mine is located in Huainan City, Anhui Province and has an annual production capacity of 4 Mt/a. The absolute gas emission of the mine in the air roadway area of 121,304 workface is 1.0~2.0 m^3^/min. It has an average dip angle of 9°. The roof is mudstone (with an average thickness of 4.2 m), the floor is sandy mudstone (with an average thickness of 3.25 m). The roadway is supported by bolt, mesh, and cable. It has a rectangular section with a section area of 17.5 m^2^. The seam is located at a depth greater than 967 m. The ground pressure is large during the driving process, the disturbance stress during the mining process of the working face is large, and the roadway is seriously deformed. It belongs to the deep mining roadway.

### 5.2. Layout and Construction of Measuring Points

During the mining process of the working face, the rock surrounding the roadway is deformed owing to the influence of mining stress, and the stress of the bolt changes. By installing the FBG force-measuring bolt on the roof and side of the roadway, and combining a sensor system, the axial force of the bolt was monitored to elucidate the law governing the change of the rock surrounding the roadway.

**Figure 9:**
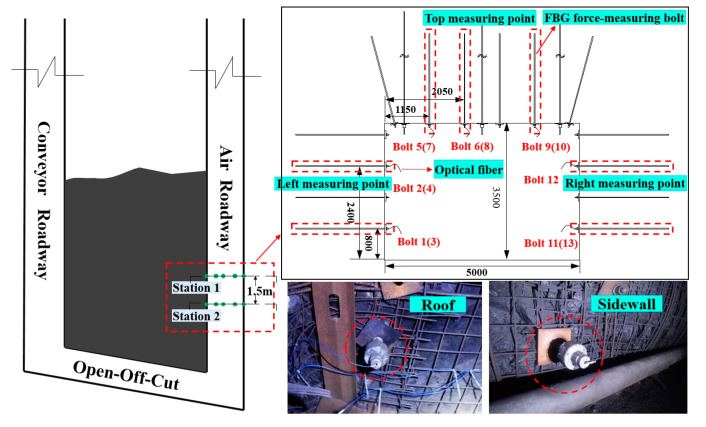
Installation diagram of FBG force-measuring bolt.

Considering the mining situation, geological characteristics, and the production plan of the working face, the FBG force-measuring bolt was arranged in a relatively flat section of the air roadway roof of the 121,304 working face. The distance between the measuring station and the stopping line of the working face was approximately 20 m. Two measuring stations and 13 force-measuring bolts were arranged in total. The distance between the measuring stations was 1.5 m. The size of the bolts was Φ20 × 2400 mm. The anchoring method was end anchoring with a length of 70 cm, and a total of five grating measuring points were located in the anchoring segment. The layout and installation scheme and effect are shown in Figure 9.

Survey station 1: the #1, #2 force-measuring bolts were arranged at the side wall (left side) of the coal mining; the #5, #6, and #9 force-measuring bolts were arranged at the roof; the #11 and #12 force-measuring bolts were arranged at the side wall (right side) of non-coal mining.

Station 2: the #3 and #4 force-measuring bolts were arranged at the side (left side) of the coal mining; the #7, #8, #10 force-measuring bolts were arranged at the roof, and the #13 force-measuring bolts (lower part of non-coal mining side) were arranged at the side (right side) of the non-coal mining.

Owing to the bad underground conditions and installation by unskilled workers, the #10 bolts (located on the right roof) were damaged during the installation process. Therefore, during the construction of bolts, attention should be paid to the protection of installation joints to prevent the bolts from being damaged during the high-speed mixing process by the bolt-drilling machine. After installation, the installation joint cover was opened, and the monitoring system was connected with a jumper and optical cable.

### 5.3. Monitoring Results

**Figure 10:**
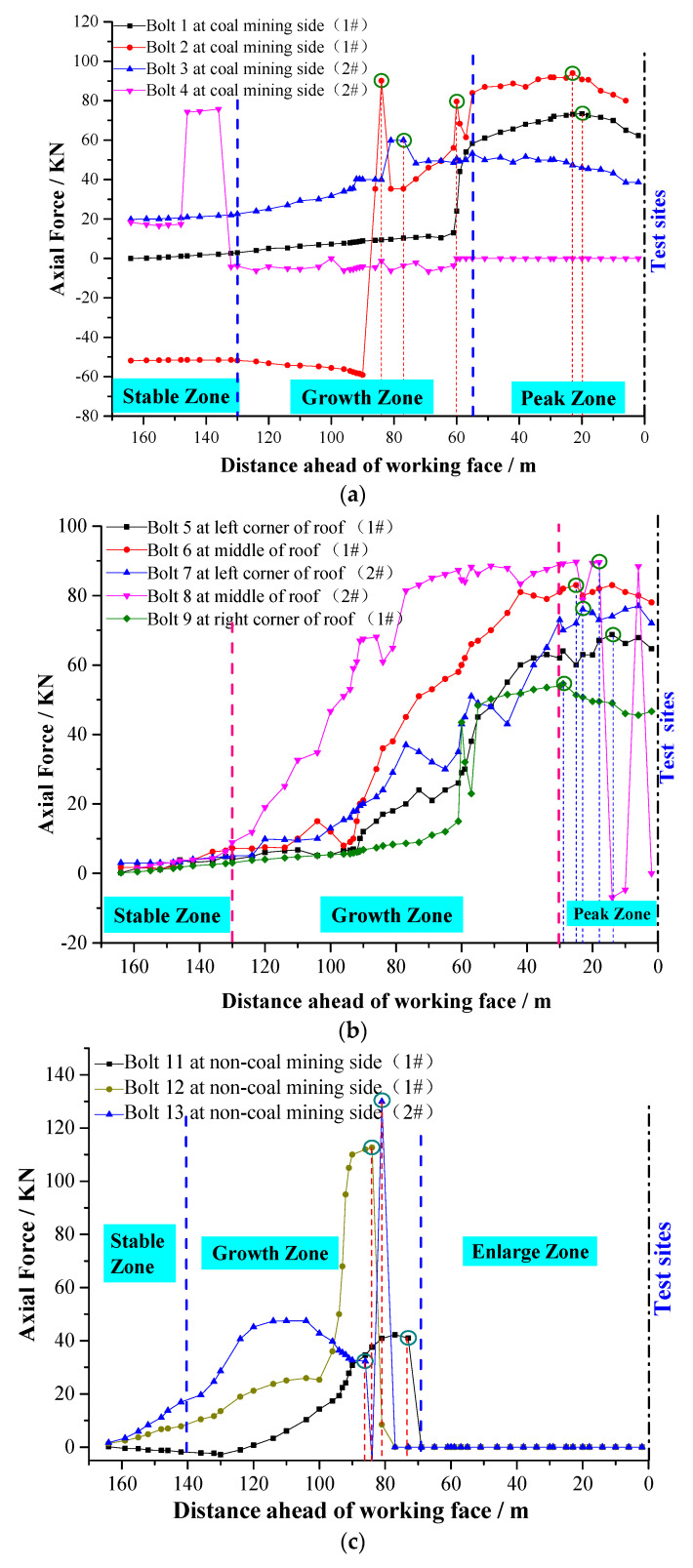

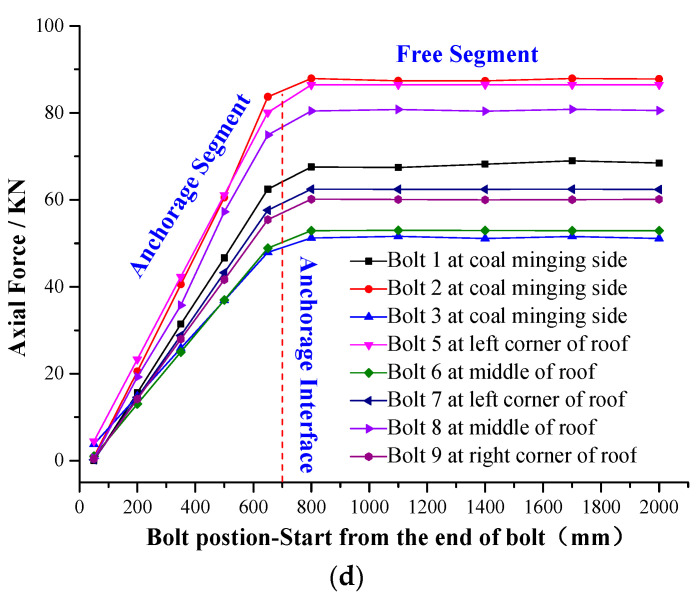
(**a**) Axial force variation curve of FBG force measuring bolt at coal mining side. (**b**) Axial force variation curve of FBG force measuring bolt at roof. (**c**) Axial force variation curve of FBG force measuring bolt at non coal mining side. (**d**) Axial force variation curve of FBG force measuring bolt at non coal mining side.

After the installation of each roadway measuring point, the monitoring system began to collect data. The monitoring began on 15 January 2017. As of 10 March 2018, the monitoring had lasted for 55 days. The working face was pushed by 180 m in total, and the real-time monitoring data of the bolt axial force of two measuring stations and three parts (coal side, roof, and non-coal side) were obtained. By sorting and analyzing the collected data, the bolt axis was obtained. The force distribution curve can be divided into two categories: horizontal and vertical. The stress condition of each bolt is compared in the horizontal direction. The stress condition of each bolt at the same position (1100 mm from the end of the bolt and 7th channel) in the entire mining process of the working face was selected for analysis. The results are presented in Figure 10a–c.

Figure 10a shows the real-time monitoring results of the axial force of the force-measuring bolt at the coal mining side. The overall change trend of the #1, #2, and #3 bolts is similar, and can be divided into three areas in total. The stable area was 130 m away from the advance working face, the axial force growth area was 55–135 m away from the advance working face, and the peak area was 55 m away from the advance working face. This is closely related to the roadway deformation and stress transfer during the mining face. With the continuous advance of the working face, the distance between the leading working face continuously decreased, while the #1 bolt pressure gradually increased. When the distance between the leading working face was 60 m, the axial force of the bolt rapidly increased. Additionally, when the distance between the leading working face was 20 m, the axial force of the bolt reached the peak value of 72.5 KN. The #2 bolt was relatively stable, and when the distance between the leading working face was 58 m and 84 m, the force of the bolt greatly fluctuated, and then the force gradually increased until the peak value of 91.8 KN at 28 m ahead of the working face. The #3 bolt stress trend exhibited a step-by-step increase, and slightly fluctuated at 78 m ahead of the working face and maximum axial force of the bolt was 51 KN. The data transmission and collection of the #4 bolt was poor. Additionally, when the distance of advance working face was 150 m, the stress of the bolt sharply changed, and sharply decreased when the distance was stable to 132 m. Data were not collected after a slight fluctuation to 60 m from the advance working face.

Figure 10b shows the real-time monitoring results of the axial force of the roof force-measuring bolt. The overall change trend of the bolt is essentially the same. The curve can be divided into three areas in total. The stable area was beyond the 130 m of the advance working face, the axial force growth area was within 30–130 m of the advance working face, and the peak area was within 30 m of the advance working face. With the continuous advance of the working face, the distance from the leading working face decreased, and the force on the bolt rod gradually increased. The axial force of the #5 bolt rod reached the peak value of 68.9 KN at 14 m of the leading working face. The peak value of the axial force of the #6 bolt was 83.3 KN when it was 25 m ahead of the working face. The peak value of the axial force of the #7 bolt was 76.3 KN at 23 m ahead of the working face. The axial force of the #8 bolt rod greatly fluctuated within 20 m of the advance working face and reached a peak value of 89.7 KN at 18 m of the advance working face. The force of the #9 bolt increased with the decrease of the distance between the advance working faces, and the force fluctuated when the distance between the advance working faces was 61 m until the peak value reached 54.6 KN when the distance between the advance working faces was 29 m.

As shown in Figure 10c, with the continuous advancement of the working face, the distance between the leading working face continuously decreased, and the stress change trend of the three #11, #12, and #13 bolts overlapped. The curve was also divided into three areas. The stable area was 140 m away from the leading working face, the axial force growth area was 68–140 m away from the leading working face, and the enlarged area was within a distance of 68 m from the leading working face. The #11 bolt was essentially unchanged in the early stage of stress, and then slowly increased. When the distance between the advance working face and the working face was 73 m, the stress decreased to zero, and data were not collected thereafter. The #12 and #13 bolts slowly increased in the early stage of stress. The #12 bolt rapidly increased at approximately 102 m of the advance working face. The #13 bolt reached the first peak at approximately 110 m of the advance working face, and then slowly decreased. When the distance between the two faces was 80 m ahead of the working face, the pressure sharply increased and then precipitously decreased, after which data were not collected.

The longitudinal direction is the stress condition of the #1, #2, #3, #5, #6, #7, #8, and #9 bolt body at different positions, simultaneously (38 m ahead of the working face; bolts #4, #10, #11, #12, and #13 were damaged during the installation or expansion construction). The results are presented in Figure 10d.

According to Figure 10d, through the stress monitoring of the entire rod of the FBG force-measuring bolt, the axial force data of 10 channels of each bolt were selected for analysis and comparison. The axial force change curve can be divided into two parts: the anchorage segment and free segment extending from the end of the anchorage. The axial force of the rod in the anchorage segment of the force-measuring bolt increased approximately linearly, and the peak value of the axial force of the bolt was concentrated on the rod. The free segment and axial force of the free segment of the bolt were essentially the same, and the axial forces for each free segment of the bolts were, approximately, as follows: 1#: 68 KN; 2#: 87 KN; 3#: 51 KN; 5#: 86 KN; 6# 53: KN; 7#: 62 KN; 8#: 80 KN; 9#: 60 KN.

### 5.4. Discussion

#### 5.4.1. Transverse Analysis of Axial Force of Bar under Multiple Stresses

**Figure 11:**
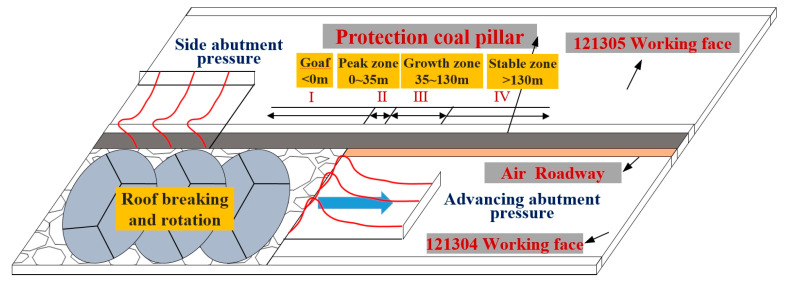
Stress distribution in mining process of working face.

According to the curve of the axial force change of the force-measuring bolt in the two above-mentioned sections, the axial force of the FBG force-measuring bolt fluctuated, and the overall fluctuation was large, which is obviously related to the strong back mining pressure in the deep roadway working face and slope instability. With the mining of the working face, “O–X” breakage occurred in the high hard roof of the roadway, which resulted in large structural movement. The rock layer collapsed and was accompanied by overturning. This released a large amount of energy, and leading and lateral supporting pressures were formed in the front and side of the coal wall, as shown in Figure 11. Additionally, the deformation speed of the deep mine roadway was fast, the deformation range of the surrounding rock was large, and the characteristics of continuous deformation and the rheology of the roadway were more obvious compared with those of a shallow tunnel. The basic roof caving coal wall and the caving belt above the goaf constantly moved forward, which also drove the influence area of the supporting pressure to move forward. Therefore, the axial force distribution curve of the two sides of the roadway and the roof bolt can be divided into three zones: the stable zone, growth zone, and peak zone (enlarged zone), as shown at the I-II-III position in Figure 11. This is essentially in agreement with the distribution law of the over front support pressure in the mining process of the working face. The change of axial force is closely related to the mechanical properties and stress state of the surrounding rock at the construction site of the bolt. After the working face is mined, the bolts at the coal mining side are constructed into the coal seam, which are mainly affected by the vertical pressure, horizontal squeezing force of the overlying rock and the pressure from the working face. The analysis shows that the rock mass in the coal mining side is softer and deforms faster, so the axial force increases relatively earlier after the working face is mined. The roof bolts are constructed into the hard rock layer, which are mainly affected by the vertical pressure of the overlying rock layer and the pressure from the working face. However, the roof forms a self-stable balanced arch structure, which has a certain self-supporting capacity, so the axial force growth duration of bolt rod is relatively long. In addition, under the influence of high ground stress and mining stress superposition, the surrounding rock of the deep well strong mining roadway often exhibited large deformation for a long time, and the occurrence frequency and strength of the roadway pressure and rock burst increased. Therefore, the axial force of the force-measuring bolt sharply increased many times and then sharply decreased.

**Figure 12:**
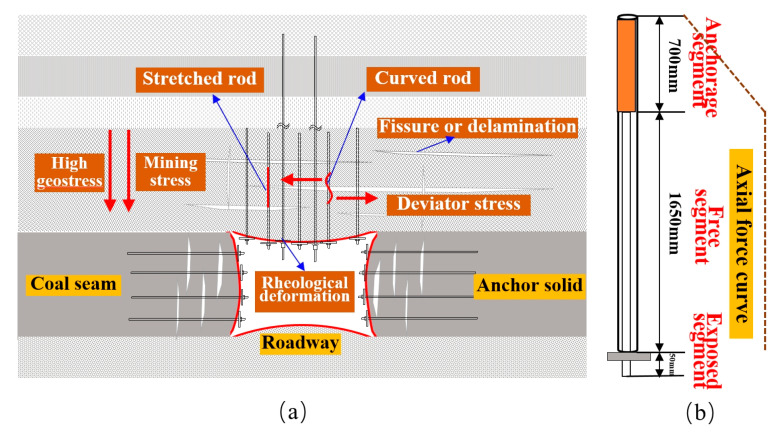
(**a**) The whole stress of bolt in mining process of working face. (**b**) Axial stress of single bolt.

According to Figure 10a–c, when the force-measuring bolt was within 130 m from the working face, its axial force began to gradually increase, indicating that it had entered the area affected by the advance bearing pressure of the working face at this time. Moreover, under the action of excavation and strong mining, the roadway was subjected to the effect of loading and unloading in different directions, which resulted in the increase of deviating stress and stress gradient in the surrounding rock of the roadway. In turn, this resulted in the partial negative value of the axial force of the bolt in the monitoring data, indicating that the bolt section was not only subjected to tensile action at this time, but also bore the shear force, which resulted in the bending of the bolt. Under the action of continuous pressure and loading and unloading, the surrounding rock stress of the roadway constantly changed, and the axial force of the bolt constantly fluctuated. The maximum axial force of the two sides and roof FBG force-measuring bolt was essentially concentrated in the range of 25–35 m ahead of the working face, which indicates that this range is the area with the higher advance supporting pressure of the working face. When the working face was close to the completion of mining, that is, when the distance between the measuring station and the working face was within 20 m, the loose circle of the surrounding rock increased, the broken rock mass in the loose circle continuously expanded, and the internal stress of the surrounding rock started being released with the increase of deformation. Additionally, the axial force of the bolt rod decreased and fluctuated to a certain extent. The advance of mining in the working face led to the formation of the fracture surface, and the rock continued to slide along the fracture surface. With rotation, sliding and other deformation occurred, and the bolt either broke or the workers damaged the optical fiber; therefore, data were not collected by the axial force monitoring of some bolts. Moreover, data were not collected when the bolt of the side wall of the non-mining face was approximately 68 m away from the working face, mainly because the advance support of the working face had to expand the non-mining side, which resulted in bolt damage, as shown in Figure 12a.

#### 5.4.2. Longitudinal Analysis of Axial Force of Bar Body under end Anchorage

The anchorage installation method of the bolt is end anchorage. Only the end of the bolt made contact with the surrounding rock through an anchorage agent. Therefore, the axial force distribution law can be divided into two parts: the anchorage segment and free segment. Because the rod body of the anchorage segment was wrapped by the anchorage agent, the axial strain at the end of the anchorage segment was close to zero. Hence, the axial force of the bolt at this location was close to zero. Because the end of the bolt extended to the exposed end, the change of the axial force in the anchorage segment increased approximately linearly. Because there existed a gap between the bolt and the drilling hole in the free segment, and the deformation separation layer of any part of the rock stratum within the anchorage range was evenly distributed to the length of the entire rod body, the strain and axial force along the length direction of the free segment of the bolt were essentially the same, as shown in Figure 12b. It is assumed that less than 1/4 of the maximum axial force of the bolt is a low-stress area, while more than 3/4 of the maximum axial force of the bolt is a high-stress area (minus the exposed length). According to Figure 10d, the average length of the low-stress area of each force-measuring bolt was 212.5 mm, accounting for 9.04% of the total length. The average length of the high-stress area was 2137.5 mm, accounting for 90.96% of the total length. The bearing range was large and could adequately transfer the prestress to the anchor solid, reduce the prestress loss, and better carry the deep surrounding rock.

The field measurement data for roadway deformation reveal that the deformation of the roof, floor, and two sides slowly increased when they were 135 m away from the working face. Additionally, the deformation speed of the roof and floor and two sides obviously increased when they were 67 m away from the working face. Within the range 18–35 m ahead of the working face, the deformation of the roadway sharply increased, which is essentially consistent with the monitoring data of the bolt’s axial force. As can be seen, compared with the traditional monitoring method, the FBG full rod axial force monitoring can reflect the stress state of the support in a more timely and effective manner, and can also accurately elucidate the law governing the roadway rock pressure appearance and determine the weak and failure positions of the roadway support in a timely manner. Hence, the feasibility of the application of the real-time axial force monitoring system based on FBG sensing technology in mine roadway monitoring is demonstrated. Moreover, the system can effectively improve the timeliness and accuracy of mining pressure data collection, and indirectly increase the reliability of the bolt support and roadway safety.

## 6. Conclusions

The following conclusions were drawn from this study:

(1) Using the ANSYS numerical simulation software, the stress and displacement of different groove bars under high confining pressure were compared and analyzed, and the trapezoidal groove was found to have superior performance. Based on the FBG sensing principle, a real-time monitoring system for the coal mine roadway bolt was built around the FBG full rod force-measuring bolt with the FBG sensor at its core.

(2) The calibration system was used to calibrate the entire force-measuring bolt of the FBG. The results reveal that there existed a good linear relationship between the center wavelength offset of the FBG and the axial force. The linearity was essentially above 0.99, and the maximum was 0.9999. The sensitivity of the FBG at different channels was obtained, and the corresponding conversion formula between the bolt and the wavelength change of the FBG under the condition of temperature compensation was presented.

(3) The monitoring system was applied to the field monitoring of mine pressure. The monitoring results reveal that the axial force of the bolt was unevenly distributed and fluctuated under the action of multiple stresses and large deformation of the surrounding rock flow in a deep and sufficiently strong mining roadway. Under the influence of the roof’s large structural movement and supporting pressure, the distribution curve of the axial force of the two sides of the roadway and the roof bolt was divided into three zones: the stable zone, growth zone, and peak zone (enlarged zone). The axial force obviously changed before and after the pressure, and the existence of the negative value indicates that the bolt bore the shear force under the action of the higher deviator stress and stress gradient. The influence range of the advance supporting pressure of the roadway was approximately 130 m in the advance working face, and the peak area was 25–35 m in the advance working face.

(4) The axial force of the anchorage segment of the end bolt linearly increased outward with the anchorage end. The axial force of the free segment was the largest and the overall stress was essentially the same. The average length of the low-stress area of the bolt was 212.5 mm, accounting for 9.04% of the total length. The average length of the high-stress area was 2137.5 mm, accounting for 90.96% of the total length. The monitoring results are essentially consistent with the actual measured data of roadway deformation, and effectively reflect the roadway pressure appearance in the process of advancing the working face in real time, verify the feasibility of the system, and provide the data basis for ensuring the safe and efficient production of deep and strong mining roadways.
